# Relationships between Bone Turnover and Energy Metabolism

**DOI:** 10.1155/2017/9021314

**Published:** 2017-06-11

**Authors:** Tânia A. P. Fernandes, Luísa M. L. Gonçalves, José A. A. Brito

**Affiliations:** ^1^Instituto Superior de Ciências da Saúde Egas Moniz (ISCSEM), Campus Universitário-Quinta da Granja, 2829-511 Monte de Caparica, Portugal; ^2^Centro de Investigação Interdisciplinar Egas Moniz (CiiEM), Campus Universitário-Quinta da Granja, 2829-511 Monte de Caparica, Portugal; ^3^Instituto de Ciências Biomédicas Abel Salazar (ICBAS), Rua de Jorge Viterbo Ferreira, No. 228, 4050-313 Porto, Portugal

## Abstract

It is well established that diabetes can be detrimental to bone health, and its chronic complications have been associated with an increased risk of osteoporotic fracture. However, there is growing evidence that the skeleton plays a key role in a whole-organism approach to physiology. The hypothesis that bone may be involved in the regulation of physiological functions, such as insulin sensitivity and energy metabolism, has been suggested. Given the roles of insulin, adipokines, and osteocalcin in these pathways, the need for a more integrative conceptual approach to physiology is emphasized. Recent findings suggest that bone plays an important role in regulating intermediary metabolism, being possibly both a target of diabetic complications and a potential pathophysiologic factor in the disease itself. Understanding the relationships between bone turnover and glucose metabolism is important in order to develop treatments that might reestablish energy metabolism and bone health. This review describes new insights relating bone turnover and energy metabolism that have been reported in the literature.

## 1. Introduction

Osteoporosis is a skeletal disorder characterized by compromised bone strength which clinical and public health importance is due to the associated fractures [1–4]. Diabetes mellitus (DM) is a metabolic disorder characterized by changes in protein and lipid metabolism, combined with chronic elevations in serum glucose [5–7]. It is well established that type 1 and type 2 DM have an effect on bone density and metabolism. Osteoporosis and diabetes are prevalent diseases with significant associated morbidity and mortality. It is important to understand the mechanisms leading to diabetes, osteoporosis, and diabetic bone pathology in order to develop treatments that might reestablish energy metabolism and bone health. The relationship between diabetes and osteoporosis is complex [8–10]. It seems that both type 1 and type 2 diabetes can be detrimental to bone health and its chronic complications have been associated with an increased risk of osteoporotic fracture [1–3]. Most biochemical markers of bone resorption are related to collagen breakdown products or to various collagen crosslinks and telopeptides [11–13]. At present, serum C-terminal peptide-bound crosslinks of type I collagen (CTX-1) have been chosen as the reference standard for bone resorption rate, whereas osteocalcin is considered a good marker of bone formation [14–16]. Also, osteocalcin is a bone-specific protein that acts as a hormone regulating glucose metabolism. Several studies have indicated that hyperglycemia induces a low turnover rate by evoking osteoblast dysfunction and suppressing serum osteocalcin levels. Values of osteocalcin were associated with glucose levels. These findings suggest that mature osteoblast function is specifically affected by glucose metabolism in type 2 diabetes (T2DM) [13, 17–19]. However, skeleton plays a key role in a whole-organism approach to physiology, such as regulation of bone mass, energy metabolism, and fertility. Consequently, the roles of leptin, serotonin, insulin, and osteocalcin in these pathways have been postulated [5, 20–23]. Incipient suggestions refer that low bone mass is detected in type 1 diabetes (T1DM) although the pathogenesis is likely to be multifactorial. Additionally, bone is metabolically active, secreting growth factors and proteins that modulate insulin sensitivity and insulin secretion [2, 3, 15]. Furthermore, osteocalcin can regulate peripheral insulin sensitivity and stimulate insulin synthesis. These findings take bone beyond its function of body support and organ protection, performing an important role in regulating intermediary metabolism, being possibly both a target of diabetic complications and a potential pathophysiologic factor in the disease itself [5, 20, 24, 25].

The insights that diabetes affects bone turnover are way more described in the literature than the inverse perspective, which we intend to do. The aim of this review is to present an overview about the possibility of disrupted bone turnover inducing changes in energy metabolism.

## 2. Endocrine Nature of Bone

Bone has been viewed as a relatively static tissue, essential for locomotion, body support, and organ protection, but it is also a regulator of numerous metabolic processes that are independent of mineral metabolism. Bone integrity and its normal function depend on other organs but may also affect them. Emerging evidence suggests that bone is metabolically active and can be considered as a true endocrine organ, revealing an integrative physiology where bone takes an important role in regulating intermediary metabolism. Indeed, bone has been considered an endocrine organ because of its capacity to secrete osteocalcin, a molecule expressed by the most osteoblast-specific gene [23, 24, 26–28]. Epidemiological studies in humans performed to analyze the relationship between osteocalcin and metabolic parameters, such as glycaemia, insulin secretion, *β*-cell proliferation, and lipid profile, have added information on the potential impact of the skeleton on energy regulation and glucose metabolism, through osteocalcin [7, 9, 23, 29].

### 2.1. Osteocalcin Secretion and Bioactivity

Osteocalcin is a small specific protein synthesized by osteoblasts containing three glutamic acid residues (13, 17, and 20 Glu in mouse and 17, 21, and 24 Glu in human) [22, 23, 30] and modified by carboxylation into gamma-carboxyglutamic acid residues, generating bone Gla protein (BGP) or osteocalcin. Gla residues are accountable for osteocalcin high affinity for hydroxyapatite, the mineral component of bone extracellular matrix (ECM) [27, 31]. Osteocalcin is detected at high concentrations as a component of the ECM [24, 30]. Being pharmacologically active, osteocalcin also acts as a hormone on glucose and fat metabolism [15, 24, 27]. After secretion, osteocalcin is stored in the bone ECM in carboxylated form (with no metabolic impact) and, to accomplish its beneficial effects on glucose metabolism, osteocalcin has to be activated. The osteoclast-mediated bone resorption promotes the low pH necessary for osteocalcin decarboxylation and activation. Studies suggest that the activation of osteocalcin depends on the coordination between bone formation by osteoblasts and bone resorption by osteoclasts. The acid pH is sufficient to decarboxylate the first GLA residue of osteocalcin (GLA13) and this undercarboxylated (GLU13) fragment of osteocalcin is in fact the active circulating fraction of osteocalcin, acting as a hormone [20, 21, 32, 33]. Undercarboxylated osteocalcin increases insulin sensitivity and secretion [27, 34–36], and serum levels of undercarboxylated osteocalcin negatively correlate with insulin resistance, obesity, and diabetes [7, 17, 37–39]. Therefore, the undercarboxylated form of osteocalcin appeared to mediate the metabolic effects of this hormone, such as increased *β*-cell proliferation, insulin secretion, insulin sensitivity [5, 16, 31], and adiponectin expression [5, 31, 40]. However, other studies in cell cultures have shown that both undercarboxylated and carboxylated forms of osteocalcin increase basal and insulin-stimulated glucose transport, whereas the effect of the carboxylated form was less clear [5, 41]. Lately, it was suggested that insulin signaling in osteoblasts is involved in the regulation of the body glucose homeostasis by stimulating osteocalcin decarboxylation indirectly, through the activation of osteoclasts [16, 24, 27–29].

Osteocalcin bioactivity is affected by insulin signaling in osteoblasts, increasing bone resorption through osteoprotegerin (Opg) downregulation [24, 25, 27]. Opg is a protein, also known as osteoclastogenesis inhibitory factor. It is a member of the TNF-receptor (tumor necrosis factor) that acts as an inhibitor of osteoclast maturation and activation [16, 33]. Thus, osteocalcin carboxylation is regulated by insulin signaling by inhibiting the expression of Opg, that promotes the activity of osteoclasts, and consequently bone resorption stimulation [16, 33, 42]. On the other hand, the acid pH of the resorptive lacunae induces osteocalcin decarboxylation [20, 24, 43, 44]. As osteocalcin stimulates insulin secretion, there is a feedback mechanism between osteocalcin and insulin activity and its negative regulators are in place to respond to these signals [21, 42, 45]. Osteocalcin expression is also directly controlled downstream of the insulin receptor by Runx2 (Runt-related transcription factor 2), a key transcription factor associated with osteoblast differentiation [42, 46, 47].

Osteocalcin, the most abundant noncollagenous bone-derived specific peptide, may stimulate adipose tissue to secrete adiponectin, an insulin-sensitizing factor [5, 25, 27, 48]. Leptin secretion by adipocytes fuels the sympathetic nervous system in the brain, signaling osteoblasts to enhance Esp (embryonic stem cell phosphatase) expression via ATF4 (activating transcription factor 4) [24, 46, 47, 49]. ATF4 is a member of a transcription factor family and is essential in many biological mechanisms, such as in the stress response, medullary hematopoiesis and bone resorption [24, 46, 48, 50]. Esp, a gene expressed in osteoblasts and encoding osteotesticular protein tyrosine phosphatase (OST-PTP), is known to antagonize insulin signaling in osteoblast for osteocalcin carboxylation [10, 24, 27, 31]. OST-PTP negatively regulates insulin receptor signaling and decreases osteocalcin activity by stimulating osteocalcin carboxylation [5, 7, 9, 21]. Since Esp ablation in mice may induce undercarboxylated osteocalcin to rise, improvement of glucose handling, and decreasing fat mass, osteoblasts might be considered as endocrine cells, controlling energy metabolism. Those animal models display an increase in *β*-cell proliferation, insulin secretion, and sensitivity that protect them from induced obesity and diabetes [5, 9, 31, 42]. These metabolic advantages are lost by the concomitant deletion of one allele of osteocalcin [5, 41, 51, 52]. Conversely, similar to osteocalcin-deficient mice, mice overexpressing Esp showed decreased levels of undercarboxylated osteocalcin, developed obesity, and insulin resistance [10, 31, 45, 53]. In addition, osteoblast-specific FoxO1 deficiency is associated with an anabolic metabolism profile due to increased osteocalcin expression and decreased expression of Esp [9, 21, 52, 53]. The transcriptional factor FoxO1 is also expressed in osteoblasts. It stimulates osteocalcin carboxylation and thus its inactivation, being a major negative mediator of insulin receptor in *β*-cells, hepatocytes, myoblasts, and adipocytes. Particularly, FoxO1 promotes gluconeogenesis in hepatocytes through specific gluconeogenic enzymes [36, 52, 53].

### 2.2. Testosterone, Glucagon-Like Peptide-1, and Bone

Recently, osteocalcin has also been found to regulate glucose homeostasis and male reproductive functions, stimulating testosterone biosynthesis in Leydig cells, via a pancreas-bone-testis axis. Testosterone is a sex steroid hormone that displays testicular functions, such as germ cell survival and spermatogenesis and other functions beyond reproduction, among which is the regulation of bone metabolism. Since endocrine regulation is liable to feedback mechanisms, the hypothesis that bone may regulate the synthesis and secretion of sex steroid hormones has been raised [54]. Thus, it has been postulated that bone, energy metabolism, and reproduction may be interrelated [22, 23]. This functional coordination may be mediated by osteocalcin receptor GPRC6A. Osteocalcin binds to Gprc6a expressed in Leydig cells of the testes, promoting cAMP production that leads to the expression of several genes encoding the enzymes that are necessary for testosterone biosynthesis [54, 55]. In *Gprc6a*, knockout mice were observed with increased fat mass, disrupted testosterone production, insulin secretion, and glucose homeostasis [56]. Insulin signaling in osteoblasts enhances osteocalcin activity, which, in turn, favors insulin secretion, contributing to the biosynthesis of testosterone in an osteocalcin-dependent way. Testosterone, on the other hand, favors bone growth, maturation, and maintenance [22, 23].

Interestingly, when orally administered, the osteoblast-derived hormone osteocalcin has the ability to indirectly regulate insulin secretion, not only through production of other insulin-regulating hormones, such as testosterone, but also by stimulation of intestinal GLP-1 secretion [56]. Glucagon-like peptide-1 (GLP-1) has been shown to regulate both bone remodeling and energy homeostasis [38]. This incretin hormone is secreted by L cells in the lower small intestine, in response to food intake. The main role of GLP-1 is to stimulate insulin secretion by pancreatic *β*-cells in a glucose-dependent manner and suppress glucagon secretion from *α*-cells. GLP-1R is a G protein-coupled receptor, playing a role in glucose metabolism. GLP-1 binds to its receptor in order to exert its biological effect, increasing intracellular cAMP and stimulating ATP production in the mitochondria and insulin secretion [57, 58].

GLP-1 receptor was found to be expressed in a variety of tissues such as the stomach, heart, lungs, kidneys, and brain [57, 59]. In addition to the pancreatic effects, reduction in body weight, delay in gastric emptying, and appetite suppression have been reported, suggesting its potential in obesity treatment. Recent studies revealed that GLP-1 might also take part in bone metabolism. Nuche-Berenguer et al. (2009, 2011) discovered that osteoblastic precursor cells express functional GLP-1 receptors and that the administration of GLP-1 in both normal and diabetic rats normalizes impaired bone structure, promotes bone formation, and restores bone mineral densities, suggesting a possible anabolic effect of GLP-1 on trabecular bone [57, 58].

It seems that GLP-1 action is more extensive than it was thought, and results reveal the potential role of this incretin in bone metabolism. T2DM patients are already starting treatments with GLP-1 mimetics [59]. Additionally, these discoveries emphasize the potential use of GLP-1/GLP-1R in diabetes-related bone defects treatments [57].

### 2.3. Other Bone-Derived Hormones

Fibroblast growth factor-23 (FGF23) is another peptide hormone that is produced by bone cells and beyond its role in mineral metabolism also acts in other tissues, primarily in the kidney [60–62]. The FGF23 is an osteocyte-derived hormone considered the major regulator of circulating phosphate levels and 1,25-dihydroxyvitamin D metabolism via a bone-kidney axis [63]. Increased serum concentrations of circulating FGF23 are observed in many hypophosphatemic disorders and it negatively impacts in chronic kidney disease, a global health problem [60, 61]. FGF23 enhances phosphate excretion into urine by restricting its absorption in the proximal renal tubule. Additionally, this hormone suppresses intestine absorption of Ca and circulating phosphate by lowering phosphate levels and reducing 1,25-dihydroxyvitamin D synthesis [62, 63]. However, in a regulatory loop, decreased phosphate and 1,25-dihydroxyvitamin D concentrations are great stimulators of FGF23 synthesis in vivo and in vitro [62], although the specific mechanisms of phosphate sensing and FGF23 secretion in osteocytes are yet to be better understood [62, 63].

Additionally, it is important to mention the newly identified role of bone-derived lipocalin 2 (LCN2) on appetite control in mice. It was demonstrated by molecular and genetic analyses in mice that LCN2 is an enriched osteoblast-derived hormone [64]. This protein was thought to be mostly produced by adipose tissue; however, it was observed that LCN2 secretion is tenfold higher in the osteoblasts than in the white adipose tissue. The authors discovered that LCN2 suppresses the appetite, suggesting a novel role for bone in metabolic regulations [64]. The investigators concluded that LCN2 is a new ligand for melanocortin 4 receptor (MC4R) in the hypothalamus, by crossing the blood-brain barrier and suppressing the MC4R-dependent anorexigenic (appetite-suppressing) pathway. Although there is further investigation ahead, these findings highlight the potential role of bone in the regulation of obesity and insulin resistance [64].

## 3. Adipose Tissue in Control of Bone Mass

Adipose tissue is a metabolically active tissue with endocrine effects. It produces cytokines and adipokines that may influence the activity of other tissues [4, 45, 65, 66]. There are distinctive types of adipose tissues (subcutaneous white adipose tissue, visceral white adipose tissue, brown adipose tissue, and bone marrow adipose tissue) with different relationships with BMD (bone mineral density) in altered bone remodeling. However, the contribution of bone marrow adipose tissue in fat-bone interface is more established. The amount of bone marrow adipose tissue is correlated to BMD loss in ageing, menopause, and anorexia nervosa and is considered a marker of compromised bone integrity [67, 68]. Adipocytes secrete leptin and adiponectin, and these adipokines have recently revealed important involvements in bone cells and control of energy homeostasis [5, 26, 69, 70]. Bone marrow mesenchymal stem cells give rise to both osteoblasts and adipocytes. In ageing process and in many secondary causes of osteoporosis, the hematopoietic bone marrow in trabecular bone is followed by progressive marrow infiltration with adipocytes and replaced by adipose tissue [20, 31, 53]. The interaction between adipose tissue and bone is notable and the adipose tissue distribution affects bone mass. It has been proven that visceral adipose tissue is a negative predictor of lumbar spine and whole body BMD in obese girls [71]. Also, high body mass index reduces fracture risk, while low body mass increases bone loss, leading frequently to osteoporosis [4, 5, 20, 27]. Thus, bone remodeling and energy metabolism are believed to be connected.

### 3.1. Leptin and Its Actions

The distribution and the excess of adipose tissue is an important factor in the induction of insulin resistance and the individual predisposition to DM. Both obesity and lipodystrophy are risk factors for the development of insulin resistance and glucose intolerance. Fat is an important source of peptides and cytokines which modulate metabolic homoeostasis [4, 69, 70]. Adipocytes affect bone and energy metabolism by secreting leptin. Leptin may influence bone metabolism through different pathways via direct and indirect actions. Leptin may improve insulin sensitivity, possibly mediated by insulin-like growth factor-binding protein 2. This fact may have positive effects on bone, proposing that leptin may directly stimulate osteoblasts function [5, 45, 69, 72]. However, leptin regulates indirectly osteoblasts through the hypothalamus by stimulating sympathetic tone and suppressing bone formation, enhancing bone resorption by decreasing osteoblast proliferation, via skeletal *β*2 adrenergic receptors on osteoblasts [31, 49, 73]. Researchers suggest that the predominant effect of leptin on the regulation of bone mass is through the central nervous system [46, 74, 75]. The neuronal mediation between leptin signaling in the brain and in osteoblasts is the SNS, through *β2* adrenergic receptors [20, 49, 73].

Leptin is an adipocyte-specific molecule, crucial in appetite regulation and bone metabolism. Its main function is to inhibit appetite and increase energy expenditure. It stimulates the sympathetic nervous system (SNS) by acting in the brain through a single receptor, and it is considered to provide the brain feedback concerning energy storage [27, 53, 76]. Over the evolution period, leptin only appears in vertebrates and it is associated with bone remodeling; thus, bone is considered as a key target for this hormone. Moreover, researchers proposed that the threshold of leptin signaling necessary to affect bone mass is lower than that needed to affect appetite [20, 73, 77, 78]. Observations in rat model experiments revealed that low doses of leptin prevented bone loss and high doses inhibited femoral growth and triggered decreased bone formation and resorption [5, 79].

### 3.2. Leptin, Serotonin, and Bone

The relationship of leptin to bone mass has proven to be complex due to the direct and indirect actions of leptin on bone. Leptin receptor (Lepr) is vastly expressed in ventromedial (VMH) and arcuate (Arc) hypothalamic neurons, and injuries in these neurons demonstrated their role in the bone and appetite regulation [5, 49, 80]. However, a novel approach suggests that leptin receptor in hypothalamic ventromedial neurons is not necessary for triggering leptin action [5, 81]. A selective inactivation of Lepr in VMH or arcuate neurons in mice fed on normal chow did not affect appetite or the increase of bone mass. Probably leptin signals other sites of the brain to regulate metabolism, as the synthesis of neurotransmitters that will later act in the hypothalamus. Serotoninergic neurons might mediate this action since leptin deficiency impacts on bone turnover; thus, metabolism can be overturned by obstructing serotonin secretion in the brainstem [5, 7, 27, 73]. Neuronal connections between serotonin-producing neurons and hypothalamic neurons may exist and possibly have essential functions, as inactivation of serotonin receptors in specific neurons of the hypothalamus resulting in osteoporosis or anorexia. The observation that individuals chronically treated with serotonin reuptake inhibitors can develop low bone mass suggested that serotonin and leptin signaling may intersect in the brain [20, 27, 82, 83]. Serotonin signaling in VMH neurons reduces the activity of the SNS, inhibiting the formation of bone mass and serotonin signaling in Arc neurons inhibiting appetite [20, 73, 84]. Leptin may regulate energy metabolism through where it signals in the brain. Serotonin suppresses catecholamine synthesis from hypothalamic neurons (actions that favor increase of bone mass), and leptin inhibits serotonin for binding serotoninergic neurons of brainstem. As a result, osteoblasts are inhibited for catecholamine binding to their beta-2 adrenergic receptors and bone formation and osteocalcin synthesis are compromised [7, 28, 73].

However, beyond acting as a neurotransmitter, serotonin is also synthesized in the gut, and there are studies suggesting there is no role of serotonin in the bone tissue through the brain, but serotonin gut-derived. Peripheral serotonin produced in the gut contributes to circulating serotonin levels and is a negative regulator of osteoblast proliferation, bone mass, and quality [85, 86]. This negative modulation on bone is thought to be mediated by serotonin receptors and transporter in osteoblasts and osteoclasts. In addition, Cui et al. suggest that serotonin can bind covalently with proteins, by transglutaminase-mediated serotonylation reaction, modifying their function [86]. These authors explain that serotonin has significant interactions with plasma fibronectin, interfering on bone extracellular matrix assembly and mineralization; hence, increased peripheral serotonin concentrations may weaken bone matrix and negatively affect bone quality and mass [86]. Furthermore, Vernejoul et al. showed that bone might gather a microserotoninergic system by synthetizing serotonin through osteoclasts that can act locally on osteoclast precursor differentiation [87].

### 3.3. Adipokines and Diabetes

Neumann et al. observed that in T1DM patients, leptin seems to be positively related with daily insulin dose, body mass index, and body fat mass, while in healthy controls, such association was not observed [45]. However, in these T1DM patients, adiponectin was higher for both males and females and appears to be inversely related with body mass index and daily insulin dose. Additionally, the study showed that, in both T1DM and control groups, total OC levels were inversely associated with body mass index. However, total OC levels were gender-dependent, with significantly lower OC levels in males with T1DM than in male controls [45]. This result is consistent with a meta-analysis, revealing that total OC levels were found to be reduced in T1DM patients [88]. Moreover, in these meta-analysis, Starup-Linde et al. realized that CTX levels were also significantly decreased in T1DM patients and that bone biomarkers of bone formation and resorption were not influenced by glucose itself but might be altered by DM unknown factors [88].

Adiponectin is an adipocyte-specific protein and a major regulator of bone mass and energy metabolism in the glucose and lipid metabolism, and it circulates in human plasma at high levels [5, 41, 66]. Adiponectin enhances insulin sensitivity by stimulating fatty acid oxidation, decreases plasma triglycerides, and improves glucose metabolism [65, 89, 90]. The degree of visceral adiposity has revealed to be inversely associated to adiponectin levels, acting as a negative predictor of adiponectin. In Japanese T2DM male patients, hypoadiponectinemia was related with visceral fat accumulation rather than subcutaneous fat [91]. Adiponectin levels are lower in obese than in thin individuals and a reduction in adiponectin expression is associated with insulin resistance. Concentrations of plasma adiponectin are negatively associated with glucose, insulin, triglyceride levels, and body mass index. Accordingly, adiponectin levels are positively related with insulin sensitivity both in healthy and diabetic individuals and insulin-stimulated glucose disposal [5, 90, 92]. Reduced expression of adiponectin has been associated with some degree of insulin resistance, indicating a role for hypoadiponectinemia in relation to insulin resistance [4, 89, 90]. Thus, in several insulin-resistant states, such as obesity, T2DM and in patients with coronary artery disease adiponectin plasma levels are reported to be reduced. Yet increased plasma adiponectin levels are reported to be related with T1DM and anorexia nervosa and higher insulin sensitivity. A decrease in plasma glucose levels and an increase in insulin sensitivity with adiponectin administration have been verified [5, 27, 92]. Moreover, adiponectin influences the development of insulin resistance and T2DM. The activity of adiponectin is associated with leptin, resistin, steroid and thyroid hormones, and glucocorticoids, among others [4, 27, 65].

### 3.4. Adiponectin and Regulations

It was observed that circulatory levels of adiponectin were negative predictors of BMD and have negative effects on trabecular bone, whereas leptin was found to be a positive predictor [5]. In mice lacking Esp, the expression and circulating levels of leptin, an insulin-sensitizing hormone, was not affected, whereas adiponectin was increased [31]. Adiponectin has the skill to stimulate insulin sensitivity and has antiatherogenic and anti-inflammatory properties; thus, adiponectin is essential for physiological and pathophysiological studies with the aim of potential therapeutic applications [65, 69, 83, 92], particularly in states associated with low plasma adiponectin levels. It may prevent also age-related bone loss by decreasing the sympathetic nervous system as a regulator of bone mass accrual [7, 66].

Adiponectin is identified for its insulin-sensitizing ability in animals fed a high fat diet. However, adiponectin absence in animals fed with a normal diet does not seem to clearly alter insulin sensitivity, suggesting that adiponectin may affect other physiological pathways than energy metabolism [66]. However, adiponectin is identified for its insulin-sensitizing ability in animals fed a high fat diet. This hormone can affect bone tissue, since during evolution adiponectin, as leptin, appear with bone and osteocalcin, may stimulate adipose tissue to secrete this hormone [66].

Several studies suggest that adiponectin regulates and applies two opposite mechanisms on bone mass formation. Also, it partly antagonizes leptin regulation of the sympathetic nervous system, inhibiting the activity of the sympathetic tone. By acting centrally, adiponectin opposes several functions of leptin. Its dual regulation occurs by decreasing FoxO1 activity [66]. Adiponectin acts directly in osteoblasts, by unknown signaling pathways, obstructing their proliferation, and enhances their apoptosis, through a PI3 kinase-FoxO1-dependent process, decreasing both bone mass and circulating osteocalcin levels. Adiponectin decreases FoxO1 activity in a PI3 kinase-dependent way in osteoblasts, accomplishing its roles, independently of its known receptors and signaling pathways [53, 66]. However, adiponectin affects bone mass accrual through a second mode of action, decreasing the activity of the sympathetic nervous system. Thus, adiponectin signals the neurons of the *locus coeruleus*, part of the brainstem, through FoxO1, to reduce the activity of the sympathetic tone, thereby increasing bone mass and decreasing energy expenditure. Hence, adiponectin signals by decreasing FoxO1 activity in osteoblasts and neurons. It appears that the adipocyte is a rare, if not a unique, example of an endocrine cell secreting two hormones with opposite effects on the same physiological mechanisms. Therefore, adipocytes seem to be of great importance in the control of bone mass accrual [50, 53, 66].

## 4. Bone, Energy Homeostasis, and Glucose Metabolism

Osteocalcin receptor GPRC6A is expressed in different tissues including the liver, skeletal muscle, brain, testis, bone, and pancreatic *β*-cells [20, 93–96]. Since the biological action of osteocalcin in the brain does not rely on GPRC6A, osteocalcin might have other undescribed physiological functions [7, 28]. Glucose metabolism can be regulated by undercarboxylated osteocalcin directly, throughout the receptor GPRC6A, increasing pancreatic *β*-cells proliferation and enhancing insulin synthesis and secretion [7, 10, 23, 51]. Osteocalcin enhances energy expenditure and insulin sensitivity via other mechanisms. Osteocalcin increases adiponectin expression in white fat and reduces lipid accumulation and inflammation in steatotic liver, and these mechanisms may distress insulin sensitivity [19, 21, 24]. Furthermore, osteocalcin controls the expression of genes implicated in energy expenditure in brown adipose tissue [19, 24, 25] and skeletal muscle. It also increases mitochondrial biogenesis in the muscle, stimulating energy consumption [19, 24, 31, 51].

The outcome that energy metabolism affects bone turnover suggested that an endocrine feed-forward loop should exist [24, 27, 31, 45]. The existence of a reciprocal regulation between bone and energy metabolisms is supported by a growing number of evidence [7, 28]. Bone remodeling occurs continuously and simultaneously in several skeleton areas of the body, and this physiological function is highly dependent on the energetic status of the organism, requiring large energy consumption. Therefore, bone and energy metabolisms are unavoidably linked, and a coordinated endocrine regulation of bone and energy metabolism must exist, where hormones have been implicated in the control of bone remodeling [5, 24, 27, 33]. As a result, impairments in glucose metabolism may distress the skeleton [5, 27, 43].

### 4.1. Bone and Insulin Signaling

Insulin signaling in osteoblasts might regulate whole body glucose homeostasis by controlling osteocalcin activation. Hence, osteocalcin bioavailability is increased by insulin signaling in osteoblasts through stimulating bone resorption [16, 24, 42]. Mice lacking insulin receptor (InsR) showed compromised insulin sensitivity, insulin secretion, and glucose tolerance. Also, these mice accumulate more fat than those in control groups and revealed reduced energy expenditure and serum levels of undercarboxylated osteocalcin [16, 24, 42, 44]. With respect to the former, correlation concerning fat and bone is assorted in overall energy metabolism due to its paracrine and endocrine functions, and osteocalcin, leptin, and adiponectin are essential hormones in bone remodeling process [5, 24, 27]. However, bone and pancreas are also related in the control of energy metabolism and bone mass, being insulin and undercarboxylated osteocalcin the biological molecules involved in this endocrine path. Osteocalcin regulates energy metabolism, in part, by enhancing insulin secretion by pancreatic *β*-cells [16, 31, 42, 66]. In a feedback loop, insulin signals back in osteoblasts to increase osteocalcin activity and thus insulin secretion [16, 42, 66]. Osteocalcin also signals in adipocytes, where it favors the synthesis of adiponectin, improving glucose metabolism by stimulating the adipocyte sensitivity to insulin and insulin secretion [5, 31, 66].

Recent studies suggest that bone may as well control insulin sensitivity given that the deletion of insulin receptor in osteoblasts conducts to insulin resistance and obesity. Osteoblasts express the insulin receptor and when activated by insulin promotes osteoclast resorption, providing osteocalcin undercarboxylation [5, 16, 20, 42]. Since the deletion of the insulin receptor in muscle was not associated before with alterations in blood glucose, serum insulin, and glucose tolerance, bone may be the tissue involved in insulin and glucose regulation circuit [20, 26, 42]. The metabolic disorders associated with the deletion of the insulin receptor are rectified by osteocalcin administration. Besides, mice lacking insulin receptor in the osteoblast demonstrated an osteopenic phenotype. These observations propose that insulin and undercarboxylated osteocalcin are the molecules implicated in bone and pancreas interconnection for the control of energy metabolism and bone mass [5, 10, 19, 28].

### 4.2. Osteokines and Glucose Metabolism

Another study found that bone has a crucial assignment in the control of energy metabolism through osteocalcin and osteoblasts. The findings suggest that, underneath the control of glucose metabolism, a bone-derived factor other than osteocalcin must be implicated [29]. Actually, there are already studies discussing the role of NPY coming from the bone tissue or of sclerostin as other osteokine which could also play a role in glucose metabolism, needing further investigation [97–99]. The study conducted by Yoshikawa et al. [29] issued that similar to the phenotype observed in osteocalcin-deficient mice, partial ablation of osteoblasts in adult mice fallouts in metabolic abnormalities such as hypoinsulinemia, hyperglycemia, glucose intolerance, and decreased insulin sensitivity, mainly reversed by undercarboxylated osteocalcin injection. However, osteoblast ablation also decreased gonadal fat and increased energy expenditure, unlike osteocalcin suppression [29]. Additionally, Sato et al. [100] recently revealed that osteocyte-null mice shows adipose tissue atrophy and decreased total osteocalcin, suggesting the implication of another osteocyte-derived factor in these phenotypes. Osteocytes are mature bone cells derived from osteoblasts that are actively involved in local bone turnover, through mechanosensory mechanisms. Together, these observations suggest that additional osteoblast or osteocyte-derived factors might be implicated in the endocrine regulation of glucose metabolism by bone [100].

Regarding the effect of circulating levels of insulin and osteocalcin on bone turnover, published studies showed that biomarkers of bone resorption positively links with insulin sensitivity/secretion and serum levels of undercarboxylated osteocalcin [15, 33, 101]. Moreover, Lacombe et al. [33] demonstrated that glucose intolerance negatively links with the serum levels of undercarboxylated osteocalcin, supporting the idea that the activation of osteocalcin in vivo is necessary to a suitable bone. The authors suggested that proper bone resorption by osteoclasts is required for the partial decarboxylation and activation of osteocalcin in vivo and determinant for glucose metabolism in mice. These authors realized that an increased number of osteoclasts induced augmented serum levels of undercarboxylated osteocalcin and improved glucose tolerance [33]. Furthermore, pharmacological inhibition of bone resorption or osteoclast ablation seems to be associated with reduced serum levels of undercarboxylated osteocalcin and compromised glucose tolerance [16, 33, 42].

### 4.3. Osteoblast-Specific FoxO1, Opg Expression, and Glucose Metabolism

The Forkhead transcription factor FoxO1, through its expression in osteoblasts and due to its ability to suppress the activity of osteocalcin by stimulating osteocalcin carboxylation, reduces *β*-cell proliferation and insulin secretion [46, 52, 53] and decreases insulin sensitivity in insulin-target tissues such as adipose tissue, the liver, and the muscle, increasing blood glucose levels and hence affecting energy expenditure and glucose metabolism. Interestingly, in a feedback mechanism of regulation, insulin signaling suppresses FoxO1 activity in osteoblasts, stimulating the activity of osteocalcin [16, 46, 52, 53]. In a whole-organism approach to physiology, FoxO1 can regulate glucose homeostasis by its linkage among skeleton, pancreas, and insulin target organs through regulation of insulin actions and osteocalcin activity [29, 31, 42, 53]. An in vitro study carried out by Ferron et al. revealed that FoxO1 controls Opg expression in osteoblasts and suppression of Opg expression by insulin signaling, inducing FoxO1 phosphorylation which results in its nuclear export [16, 24]. Accordingly, in an experimental study in mice lacking FoxO1, only in osteoblasts, mice displayed a reduced level of Opg in bone and an increased number of osteoclasts, amplified *β*-cell proliferation, insulin secretion, and insulin sensitivity [52]. This study, developed by Rached et al. [52], also reported that serum levels and expression of adiponectin were amplified. Moreover, the expression of osteocalcin increased 50%, the circulatory levels of osteocalcin increased 30%, and the Esp expression was reduced 75%. However, the removal of a single osteocalcin allele from mice lacking FoxO1 resulted in a complete reversal of the metabolic abnormalities of the previous data [52]. Therefore, these findings support the hypothesis of glucose homeostasis regulation by osteoblasts [5, 52, 102].

Another study conducted by Rached et al. [102] demonstrated that FoxO1 is essential for proliferation and redox balance in osteoblasts, controlling bone formation. Osteoporosis is associated with reduced number of osteoblasts and increased levels of oxidative stress in these cells and the FoxO1 assigns stress resistance. FoxO1 regulation of osteoblast proliferation occurs because of its interaction with ATF4 [102]. In mice lacking FoxO1 in osteoblasts, reducing oxidative stress levels or increasing protein intake regularizes bone formation and bone mass. These data find FoxO1 as an essential regulator of osteoblast physiology and a connection between oxidative stress and the regulation of bone remodeling [102]. The transcription factor ATF4 plays a role in glucose metabolism and insulin sensitivity by acting through osteoblasts [46, 47]. ATF4 is necessary to directly induce Esp expression in osteoblasts [24, 47, 103]. Experimental studies with mice with ATF4 ablation in osteoblasts or in all tissues revealed improved glucose tolerance, insulin secretion, and insulin sensitivity in the liver, fat, and muscle, likewise with mice lacking FoxO1 [24, 47, 104]. It was suggested that ATF4 forms dimers with other transcription factors, including FoxO1 in osteoblasts. Kode et al. [46] proposed that ATF4 and FoxO1 synergistically combined in this functional complex induce Esp expression in osteoblasts, by directly binding to the Esp promoter and subsequently, promote glucose intolerance and compromise insulin production and sensitivity. These results establish that FoxO1 and ATF4 in bone regulate glucose homeostasis.

Furthermore, osteoclast gain-and-loss function models suggest that osteoclasts take part in bone's endocrine role and that controlling the number of osteoclasts or its activity affect glucose metabolism. The lack of Opg in mice results in an intensified bone resorption leads to an amplified number of active osteoclasts, increased undercarboxylated osteocalcin, and glucose tolerance. However, there is a decrease in undercarboxylated osteocalcin and glucose tolerance in the absence of osteoclasts in mice. In this last animal model, a decrease in *β*-cell mass and insulin secretion is also observed [21, 24, 33]. These findings suggest that variations in osteoclastic activity modulate undercarboxylated osteocalcin and indicate that osteoclasts control glucose metabolism, leastwise for endorsing osteocalcin decarboxylation and activation in vivo. Although it should not be rejected that bone resorption might regulate glucose metabolism independently of osteocalcin.

Further investigation in physiological functions may lead to the discovery of other tissues' interconnections, enabling an enhanced understanding of the pathogenesis of these diseases and the development of conceivable therapies.

## 5. Conclusions

Besides its predictable functions of body support and locomotion, bone is now considered to play an important role in a whole-organism approach to physiology. In fact, bone has proven to be an endocrine organ since it is metabolically active by secreting osteocalcin, a bone-specific protein associated to the energy and glucose homeostasis.

Bone has the ability to constantly renew itself, representing a key function on skeleton by repairing macro- and microinjuries. Published results indicate that bone remodeling and appetite might be regulated by the same hormones since low body mass index enhances bone loss and usually contributes to osteoporosis. Findings also suggest that bone resorption is determinant for glucose metabolism. An increasing number of osteoclasts are related with increased bone resorption, increased serum levels of undercarboxylated osteocalcin, and improved glucose tolerance. Additionally, a recent study showed new evidence of osteoblast-mediated glucose homoeostasis, proposing that osteoblasts have a crucial role in FoxO1-mediated actions on insulin and therefore in control of glucose homoeostasis by enhanced *β*-cell proliferation, insulin secretion, and insulin sensitivity.

Moreover, the interaction between adipose tissue and bone is significant. Adipokines such as leptin and adiponectin, secreted by adipocytes, control energy homeostasis, but also have complex actions on bone cells. Leptin, a molecule involved in appetite regulation and energy metabolism is considered to provide the brain feedback concerning energy storage. Actually, leptin controls osteoblast function system and osteoblasts control energy metabolism through osteocalcin. Actually, leptin affects insulin secretion by decreasing osteocalcin bioactivity. A common regulatory link between bone remodeling and energy metabolism has been established. Adiponectin appears to be a major regulator of bone mass and energy metabolism. In addition to its capacity to decrease activity of the sympathetic nervous system, thereby increasing bone mass and decreasing energy expenditure, adiponectin also acts directly on osteoblasts, inhibiting its proliferation and favoring its apoptosis. Hence, adiponectin regulates and applies two opposite mechanisms on bone mass accrual and partly antagonizes leptin regulation of the sympathetic tone. These results revealed that besides a cross regulation between the skeleton and energy metabolism, there is also a very tight functional relationship between fat cells and osteoblasts. Summing up, osteocalcin and insulin rise each other's activities or secretion in a feed-forward loop. Insulin acts positively on osteocalcin production and activation, which increases *β*-cell proliferation, insulin secretion, and adiponectin production on adipose tissue, enhancing peripheral insulin sensitivity, whereas other hormones, including leptin and glucocorticoids, negatively regulate osteocalcin activity by suppressing osteoblast function, osteocalcin secretion, and energy expenditure. Therefore, osteocalcin has been associated to the regulation of glucose and energy homeostasis.

Novel insights have emerged a new perspective in which skeleton plays a key role in the integrative approach to physiology associated with energy homeostasis and glucose metabolism. Future research on bone function shall clarify if it has potential therapeutic implications in diabetes, obesity, metabolic syndrome, and identify new targets for treatment.
